# 多发性骨髓瘤自体造血干细胞移植前淋巴细胞亚群与移植后造血重建及复发的相关性

**DOI:** 10.3760/cma.j.cn121090-20260115-00028

**Published:** 2026-06

**Authors:** 露露 张, 福润 安, 蓓蓓 谢, 延生 汪, 金晓 侯, 慧 秦, 毅 董, 志敏 翟, 莉莉 陶

**Affiliations:** 安徽医科大学第二附属医院血液内科，合肥 230601 Department of Hematology, The Second Affiliated Hospital of Anhui Medical University, Hefei 230601, China

## Abstract

回顾性分析2020年7月至2025年8月于安徽医科大学第二附属医院接受自体造血干细胞移植（auto-HSCT）的57例多发性骨髓瘤（MM）患者的临床资料，流式细胞术检测移植前外周血淋巴细胞亚群。多因素分析显示，较高的回输CD34^+^细胞数量（*HR*＝0.739，*P*＝0.043）与移植前较高的调节性T细胞比例（*HR*＝0.728，*P*＝0.009）是粒系快速植入的独立良好预后因素；较高的回输CD34^+^细胞数量（*HR*＝0.849，*P*＝0.042）与移植前较高的NK细胞计数（*HR*＝0.991，*P*＝0.038）是巨核系快速植入的独立良好预后因素。中位随访9.9（0.5～55.0）个月，移植前较高的CD4^+^ T细胞百分比/CD8^+^ T细胞百分比是无进展生存的独立危险因素（*HR*＝1.627，95％*CI*：1.159～2.284，*P*＝0.005）。本研究显示，MM患者auto-HSCT前淋巴细胞亚群与造血重建速度及复发风险相关。

## Abstract (en)

The clinical data of 57 patients with multiple myeloma（MM）who underwent autologous hematopoietic stem cell transplantation（auto-HSCT）at the Second Affiliated Hospital of Anhui Medical University from July 2020 to August 2025 were retrospectively analyzed. Peripheral blood lymphocyte subsets before transplantation were detected by flow cytometry. Multivariate analysis showed that a higher infused CD34⁺cell count（*HR*＝0.739, *P*＝0.043）and a higher proportion of regulatory T cells before transplantation（*HR*＝0.728, *P*＝0.009）were independent favorable prognostic factors for rapid neutrophil engraftment. A higher infused CD34⁺cell count（*HR*＝0.849, *P*＝0.042）and a higher pre-transplant NK cell count（*HR*＝0.991, *P*＝0.038）were independent favorable prognostic factors for rapid megakaryocyte engraftment. With a median follow-up of 9.9（0.5–55.0）months, a higher ratio of CD4^+^ T-cell percentage to CD8^+^ T-cell percentage before transplantation was an independent risk factor for progression-free survival（*HR*＝1.627, 95％ *CI*: 1.159–2.284, *P*＝0.005）. This study demonstrated that lymphocyte subsets before auto-HSCT are associated with the speed of hematopoietic reconstitution and the risk of relapse in MM patients.

多发性骨髓瘤（MM）是常见的血液系统恶性肿瘤，自体造血干细胞移植（auto-HSCT）仍是适合移植患者的一线巩固治疗方案[1]–[2]。尽管治疗策略不断进展，但移植后造血重建延迟及疾病复发仍是影响患者预后的关键问题[3]–[4]。近年研究提示，患者免疫状态尤其是淋巴细胞亚群分布可能在移植后免疫重建及疾病控制中发挥重要作用[5]–[6]。然而，移植前外周血淋巴细胞亚群的特征能否预测auto-HSCT后造血恢复速度及复发风险，目前尚缺乏系统研究。本研究旨在深入分析MM患者移植前淋巴细胞亚群分布特点，并探讨其与auto-HSCT后中性粒细胞、血小板植入时间及疾病复发的相关性，为优化移植策略和预后评估提供依据。

## 病例与方法

1. 病例：选取2020年7月至2025年8月安徽医科大学第二附属医院收治的57例诱导治疗后接受auto-HSCT的MM患者为研究对象，MM的诊断标准符合国际骨髓瘤工作组（IMWG）2014年诊断标准，参照IMWG疗效标准评估治疗疗效，采用DS、ISS、R-ISS及R2-ISS分期系统进行疾病分期，依据mSMART 3.0标准定义高危细胞遗传学异常。本研究经安徽医科大学第二附属医院伦理委员会批准（批件号：YX2026-231），并豁免患者知情同意书。

2. 临床资料：收集的临床资料包括患者的年龄、性别、免疫固定电泳、细胞遗传学信息、诱导化疗方案、预处理方案、回输单个核细胞（MNC）和CD34^+^细胞数、植入时间等。其中高危细胞遗传学异常指间期荧光原位杂交检测出TP53突变、1q扩增、t（4;14）、t（14;16）、t（14;20）。

3. 造血干细胞动员及预处理方案：患者疗效评估达到部分缓解（PR）以上后进行造血干细胞动员和采集。造血干细胞动员方案：25例（43.9％）采用依托泊苷联合重组人粒细胞集落刺激因子（G-CSF），18例（31.6％）采用G-CSF联合普乐沙福，7例（12.3％）采用环磷酰胺联合G-CSF，7例（12.3％）单用G-CSF动员方案。预处理方案：50例（87.7％）患者采用美法仑140～200 mg/m^2^。6例（10.5％）采用苯达莫司汀100 mg/m^2^，−4～−3 d；美法仑140 mg/m^2^，−2 d。1例（1.8％）采用白消安0.8 mg/kg，每6小时1次，−8～−6 d；依托泊苷10 mg/kg，每日1次，−5～−4 d；环磷酰胺50 mg/kg，每日1次，−3～−2 d。

4. 造血干细胞植入标准：造血重建监测的主要指标包括外周血中性粒细胞绝对计数和血小板计数。连续3 d中性粒细胞计数≥0.5×10^9^/L的首日为中性粒细胞植入时间；脱离血小板输注，连续7 d血小板计数≥20×10^9^/L的首日为血小板植入时间。

5. 外周血淋巴细胞亚群检测：采集患者外周血3～4 ml至含肝素钠的抗凝采血管中（抗凝剂与血液比例为1∶9），并立即送检进行淋巴细胞亚群分析。流式细胞术检测患者采集T细胞时外周血样本中淋巴细胞亚群的数量与比例，淋巴细胞亚群包括：CD3^+^T细胞、CD4^+^T细胞、CD8^+^T细胞、B细胞、自然杀伤（NK）细胞、CD4^+^CD25^+^CD127^high^效应性T细胞（Teff细胞）、CD4^+^CD25^+^CD127^low^调节性T细胞（Treg细胞）。具体步骤如下：在25 °C避光条件下，使用荧光标记的特异性单克隆抗体进行细胞染色15 min。染色后，加入900 µl裂解液裂解红细胞10 min，随后以400×*g*离心5 min，弃上清；用磷酸盐缓冲液洗涤细胞2次，进行流式细胞术检测。采用CytoFLEX流式细胞仪（美国Beckman Coulter公司）进行检测，数据通过CytExpert软件进行分析。CD4^+^T细胞/CD8^+^T细胞比值与Treg细胞/Teff细胞比值均基于相应细胞所占百分比计算。

6. 随访：通过查阅患者住院电子病历或电话随访，随访截止日期为2025年10月31日。总生存（OS）时间定义为自体造血干细胞回输结束至因任何原因死亡的时间，无进展生存（PFS）时间定义为自体造血干细胞回输结束至疾病复发或因疾病进展死亡的时间。

7. 统计学处理：采用SPSS 22.0和R软件4.2.1版进行数据分析和作图。分类变量用例数（百分比）描述，非正态分布的计量资料以*M*（范围）进行描述，采用Mann-Whitney *U*检验比较组间差异，移植后造血重建状态相关因素的分析采用Logistic回归模型。单因素分析中*P*≤0.05的变量纳入多因素分析。采用单因素及多因素Cox比例风险回归模型分析影响预后的独立危险因素。*P*<0.05为差异有统计学意义。

## 结果

1. 临床特征：研究共纳入57例患者，其中男34例（59.6％），女23例（40.4％），诊断时中位年龄为57（34～70）岁；IgG型27例（47.4％），IgA型11例（19.3％），IgD型3例（5.3％），轻链型15例（26.3％），不分泌型1例（1.8％）；部分患者资料缺失或未行细胞遗传学检测，DS分期Ⅰ期3例（5.4％），Ⅱ期5例（8.9％），Ⅲ期48例（85.7％）；ISS分期Ⅰ期7例（13.0％），Ⅱ期21例（38.9％），Ⅲ期26例（48.1％）；R-ISS分期Ⅰ期6例（12.0％），Ⅱ期28例（56.0％），Ⅲ期16例（32.0％）；R2-ISS分期Ⅰ期4例（8.9％），Ⅱ期15例（33.3％），Ⅲ期23例（51.1％），Ⅳ期3例（6.7％）；mSMART 3.0风险分层18例（39.1％）标危，28例（60.9％）高危。共47例患者行骨髓瘤高危遗传学检测，其中19例（40.4％）存在1q21扩增，5例（10.6％）存在t（4;14），3例（6.4％）存在P53突变，1例（2.1％）存在t（14;20），1例（2.1％）存在t（14;16），7例（14.9％）为双打击。

auto-HSCT前所有患者均接受化疗，患者中位化疗6（3～15）个周期，其中44例（77.2％）患者采用含硼替佐米和（或）来那度胺的方案联合治疗，13例（22.8％）患者采用含达雷妥尤单抗的联合方案治疗。移植前38例（66.7％）获得完全缓解（CR），13例（22.8％）获得非常好的部分缓解（VGPR），5例（8.8％）获得PR，1例（1.8％）疾病进展（PD）。auto-HSCT回输MNC中位数为7.70（1.39～34.41）×10^8^/kg，回输CD34^+^细胞中位数为5.28（1.71～34.19）×10^6^/kg。移植后中性粒细胞植入中位时间为10（9～23）d，血小板植入中位时间为10（4～22）d。所有患者移植后造血功能均顺利重建，无移植相关死亡。

2. 移植前淋巴细胞亚群特点：在可评估的54例患者中，总T淋巴细胞、CD4^+^T细胞、CD8^+^T细胞、总B淋巴细胞、NK细胞、Teff细胞、Treg细胞百分比中位数分别为84.3％（55.6％～98.6％）、45.9％（17.7％～70.7％）、36.1％（10.3％～75.9％）、3.0％（0.3％～38.2％）、9.1％（0.4％～34.3％）、5.7％（0.9％～28.4％）、7.6％（2.1％～16.1％）；总T淋巴细胞、CD4^+^T细胞、CD8^+^T细胞、总B淋巴细胞、NK细胞计数中位数分别为716.5（258.0～1 774.0）/µl、405.0（124.0～985.0）/µl、314.5（51.0～1 185.0）/µl、29.5（2.0～466.0）/µl、78.5（4.0～413.0）/µl；CD4^+^T细胞/CD8^+^T细胞比值、Treg细胞/Teff细胞比值的中位数分别为1.3（0.2～6.3）、1.3（0.2～7.2）。

3. 移植前淋巴细胞亚群与造血植入的相关性：患者接受auto-HSCT后，不同粒系植入时间（≤10 d对>10 d）和巨核系植入时间（≤10 d对>10 d）的淋巴细胞亚群比较见附表1（**扫描本文首页二维码查阅**）。粒系植入时间>10 d组的中位Treg细胞百分比显著低于植入时间≤10 d组（4.6％对8.1％，*P*＝0.017）。与巨核系植入时间>10 d组相比，植入时间≤ 10 d组的中位CD8^+^T细胞百分比显著降低（30.9％对41.5％，*P*＝0.047），中位NK细胞绝对计数显著升高（133.0/µl对57.0/µl，*P*＝0.012），中位Treg细胞百分比有升高趋势（8.0％对6.3％，*P*＝0.084）。粒系与巨核系不同植入时间组不同淋巴细胞亚群表达水平的聚类热图见附图1（**扫描本文首页二维码查阅**）。

4. 移植后造血重建状态的影响因素：分别研究患者年龄、移植前疗效≥VGPR、回输CD34^+^细胞数量、回输MNC数量、粒细胞缺乏（粒缺）伴发热时间、移植前淋巴细胞亚群对粒系和巨核系造血重建的影响。单因素分析显示，移植前Treg细胞百分比（*P*＝0.024）和回输CD34^+^细胞数量（*P*＝0.034）对粒系植入有显著影响，较长的粒缺伴发热时间有延长粒系植入时间的趋势（*P*＝0.067）；移植前NK细胞计数（*P*＝0.015）、回输CD34^+^细胞数量（*P*＝0.019）和粒缺伴发热时间（*P*＝0.028）对巨核系植入有显著影响（表1）。多因素分析显示，回输CD34^+^细胞数量（*P*＝0.043）与移植前Treg细胞百分比（*P*＝0.009）是粒系造血重建状态的独立影响因素；回输CD34^+^细胞数量（*P*＝0.042）与移植前NK细胞计数（*P*＝0.038）是巨核系造血重建状态的独立影响因素（表1）。

**表1:** 多发性骨髓瘤患者自体造血干细胞移植后造血重建时间的影响因素分析

指标	粒系				巨核系			
	单因素分析		多因素分析		单因素分析		多因素分析	
	*OR*（95％ *CI*）	*P*值	*OR*（95％ *CI*）	*P*值	*OR*（95％ *CI*）	*P*值	*OR*（95％ *CI*）	*P*值
粒缺伴发热时间	1.325（0.981～1.791）	0.067			1.398（1.037～1.886）	0.028		
年龄	0.998（0.935～1.066）	0.961			0.986（0.927～1.049）	0.657		
移植前疗效≥VGPR	0.400（0.043～3.700）	0.419			0.609（0.102～3.627）	0.586		
总T淋巴细胞百分比	1.015（0.959～1.073）	0.613			1.039（0.983～1.097）	0.175		
CD4^+^T细胞百分比	1.003（0.959～1.049）	0.889			0.983（0.942～1.026）	0.430		
CD8^+^T细胞百分比	1.007（0.969～1.046）	0.731			1.031（0.991～1.071）	0.127		
总B淋巴细胞百分比	0.986（0.898～1.083）	0.771			0.971（0.887～1.063）	0.525		
NK细胞百分比	0.985（0.919～1.056）	0.670			0.957（0.894～1.025）	0.211		
总T淋巴细胞计数	1.107（0.729～1.681）	0.632			0.856（0.558～1.313）	0.477		
CD4^+^T细胞计数	0.999（0.997～1.001）	0.178			1.000（0.998～1.001）	0.587		
CD8^+^T细胞计数	0.998（0.995～1.001）	0.264			0.997（0.993～1.000）	0.057		
CD4^+^T细胞百分比/CD8^+^T细胞百分比	0.999（0.996～1.001）	0.364			1.001（0.999～1.003）	0.481		
总B淋巴细胞计数	0.996（0.987～1.006）	0.443			0.996（0.988～1.004）	0.360		
NK细胞计数	0.996（0.988～1.003）	0.255			0.990（0.981～0.998）	0.015	0.991（0.982～0.999）	0.038
Treg细胞百分比	0.806（0.668～0.971）	0.024	0.728（0.574～0.923）	0.009	0.981（0.873～1.102）	0.751		
Teff细胞百分比	0.895（0.761～1.053）	0.181			0.880（0.751～1.030）	0.112		
Treg细胞百分比/Teff细胞百分比	0.775（0.424～1.420）	0.410			0.823（0.492～1.377）	0.459		
回输CD34^+^细胞数量	0.795（0.644～0.982）	0.034	0.739（0.552～0.990）	0.043	0.821（0.696～0.968）	0.019	0.849（0.726～0.994）	0.042
回输MNC数量	0.986（0.906～1.072）	0.735			0.983（0.910～1.062）	0.670		

**注** VGPR：非常好的部分缓解；NK细胞：自然杀伤细胞；Treg细胞：调节性T细胞；Teff细胞：效应性T细胞；MNC：单个核细胞

5. 移植前淋巴细胞亚群对疾病复发的影响：至随访结束，共10例（17.5％）患者复发，2例（3.5％）死亡，中位随访时间为9.9（0.5～55.0）个月，中位OS时间和PFS时间未达到。单因素Cox风险回归显示，CD4^+^T细胞/CD8^+^T细胞比值升高（*HR*＝1.660，95％ *CI*：1.179～2.337，*P*＝0.004）是PFS的不良预后因素，CD4^+^T细胞百分比升高也与较差的PFS相关（*HR*＝1.056，95％ *CI*：1.001～1.114，*P*＝0.045）（表2）。多因素分析显示，CD4^+^T细胞/CD8^+^T细胞比值升高是PFS的独立不良预后因素（*HR*＝1.627，95％*CI*：1.159～2.284，*P*＝0.005）（表2）。

**表2:** 多发性骨髓瘤患者自体造血干细胞移植后复发的影响因素分析

指标	单因素分析		多因素分析	
	*HR*（95％ *CI*）	*P*值	*HR*（95％ *CI*）	*P*值
年龄	1.052（0.971～1.140）	0.214		
总T淋巴细胞百分比	1.008（0.951～1.068）	0.794		
CD4^+^T细胞百分比	1.056（1.001～1.114）	0.045		
CD8^+^T细胞百分比	0.968（0.923～1.016）	0.188		
总B淋巴细胞百分比	0.856（0.708～1.034）	0.106		
NK细胞百分比	1.064（0.985～1.148）	0.113		
总T淋巴细胞计数	0.999（0.997～1.001）	0.363		
CD4^+^T细胞计数	1.001（0.997～1.005）	0.624		
CD8^+^T细胞计数	0.997（0.993～1.001）	0.189		
CD4^+^T细胞百分比/CD8^+^T细胞百分比	1.660（1.179～2.337）	0.004	1.627（1.159～2.284）	0.005
总B淋巴细胞计数	0.987（0.970～1.004）	0.125		
NK细胞计数	1.003（0.997～1.010）	0.291		
Treg细胞百分比	0.989（0.825～1.186）	0.906		
Teff细胞百分比	1.042（0.896～1.212）	0.591		
Treg细胞百分比/Teff细胞百分比	0.762（0.366～1.589）	0.468		
回输CD34^+^细胞数量	0.904（0.743～1.099）	0.310		
回输MNC数量	0.943（0.837～1.062）	0.332		

**注** NK细胞：自然杀伤细胞；Treg细胞：调节性T细胞；Teff细胞：效应性T细胞；MNC：单个核细胞

## 讨论

auto-HSCT是MM患者获得深度缓解和长期生存的关键巩固治疗[7]–[8]。然而，患者移植后造血重建的速度存在差异，且疾病复发仍是影响远期疗效的核心问题[9]–[10]。除造血干细胞数量与质量等传统因素外，移植前宿主的免疫微环境状态对移植结局的潜在影响日益受到重视[11]。

本研究通过系统分析MM患者auto-HSCT前外周血淋巴细胞亚群分布，探讨其对中性粒细胞与血小板植入时间及患者生存的预测价值。结果显示，回输CD34^+^细胞数量与粒系及巨核系植入时间相关，移植前较高的Treg细胞比例和NK细胞计数分别与更快的粒系和巨核系植入独立相关，而较高的CD4^+^T细胞/CD8^+^T细胞比值是预测移植后疾病复发的独立预后因素。

本研究中，CD34^+^细胞数量是影响粒系与巨核系植入时间的独立因素。CD34^+^细胞是造血干细胞和祖细胞的标志，其输注数量直接与移植后重建完整造血系统的潜力相关[12]。研究表明，CD34^+^细胞输注剂量与造血重建速度密切相关[13]。既往关于CD34^+^细胞数量对细胞植入影响的研究结论不一，早期Desikan等[14]发现，CD34^+^细胞数量对粒系植入无影响，与巨核系植入显著相关。Klaus等[15]则发现CD34^+^细胞数量能全面促进粒系和巨核系重建。这些研究结论的差异可能与患者人群、动员方案、移植前治疗（如新型诱导治疗药物）及时代背景相关。值得注意的是，Pasvolsky等[3]的大样本回顾性研究显示，在接受一线移植的2 479例MM患者中，高CD34^+^细胞组（>2.5×10^6^个/kg）粒系和巨核系植入时间更短。其为CD34^+^细胞数量的关键作用提供了当前临床背景下的有力证据，进一步支持了本研究的结论。

Treg细胞在auto-HSCT中已被证实可通过抑制过度炎症和移植物抗宿主病间接促进造血干细胞植入[16]。其潜在机制包括调控炎症微环境及分泌再生因子（如双调蛋白）支持组织修复[17]。然而，在auto-HSCT背景下，Treg细胞与造血重建的直接临床关联证据有限。本研究发现，MM患者移植前较高Treg细胞比例与粒系植入时间更短独立相关。有研究提示，移植前的化疗暴露会显著改变造血干细胞的克隆演化[18]，因此Treg细胞可能通过调节此类治疗后的骨髓损伤与炎症微环境间接影响造血重建，其具体机制有待阐明。

NK细胞作为固有免疫系统的核心淋巴细胞，不仅直接介导细胞毒性作用，还能通过分泌多种细胞因子（如γ干扰素、粒-巨噬细胞集落刺激因子等）广泛参与免疫调节与造血微环境调节[19]–[21]。NK细胞可能通过分泌的细胞因子间接支持巨核系祖细胞的分化与成熟，促进血小板生成[22]–[23]。本研究发现，MM患者auto-HSCT前较高的NK细胞计数是预测血小板植入更快的独立良好预后因素，可能提示移植前较好的固有免疫功能状态有利于为造血干/祖细胞的归巢、存活及分化营造更有利的初始微环境[24]–[25]。

CD4^+^T细胞/CD8^+^T细胞比值是衡量免疫平衡状态的关键指标，其降低常提示免疫功能受损并与不良预后相关[26]。对于异基因造血干细胞移植，CD4^+^T细胞/CD8^+^T细胞比值已被证实与移植后疾病复发风险相关[27]。在auto-HSCT背景下，该比值与疾病复发风险相关性的研究证据仍然有限。本研究发现移植前较高的CD4^+^T细胞/CD8^+^T细胞比值是PFS的独立危险因素。

本研究尚存在局限性。首先，作为单中心、回顾性研究，样本量有限，且患者诱导治疗方案等存在异质性，可能产生选择偏倚。其次，移植后免疫重建动态监测数据不足，未能动态观察其移植前后变化。最后，中位随访时间较短，长期生存数据有待进一步更新。

综上所述，本研究提示，移植前的CD34^+^细胞数量与淋巴细胞亚群特征共同影响MM患者auto-HSCT的结局。CD34^+^细胞是造血重建的基石，而Treg细胞和NK细胞作为患者免疫微环境的指标，可提供额外的预测价值。CD4^+^T细胞/CD8^+^T细胞比值则是与复发风险相关的独立预后因素，为优化auto-HSCT策略提供了新依据。

## Supplementary Material


